# An Unusual Cluster of Urinary Tract Infections, Including Salmonella spp., in an Iranian Ulcerative Colitis Cohort Treated With Vedolizumab: A Case Series and Comparative Analysis

**DOI:** 10.1002/jgh3.70355

**Published:** 2026-02-23

**Authors:** Yousef VatanParast

**Affiliations:** ^1^ Department of Medical Genetics, Shafa Hospital Ahvaz Iran

**Keywords:** drug safety, gut–urinary axis, Iran, real‐world evidence, salmonella, ulcerative colitis, urinary tract infection, vedolizumab

## Abstract

**Background:**

Vedolizumab, a gut‐selective α4β7 integrin antagonist, is widely used for the treatment of ulcerative colitis (UC) and is generally considered to have a favorable systemic infection safety profile. During routine clinical practice, we observed an unexpected clustering of urinary tract infections (UTIs) among patients initiating vedolizumab in an Iranian cohort, prompting further evaluation.

**Methods:**

This single‐center, observational case series included 15 adult patients with moderate‐to‐severe UC treated with vedolizumab and followed prospectively for 12 weeks for the occurrence of UTIs. A contemporaneous comparator cohort of 30 UC patients treated with infliximab (*n* = 15) or tofacitinib (*n* = 15) was followed over the same period. The primary outcome was symptomatic, culture‐proven UTI, defined as > 10^5^ CFU/mL of a single uropathogen.

**Results:**

Patients treated with vedolizumab demonstrated significant clinical improvement, with a mean Mayo score reduction from 8.2 to 3.5 during follow‐up. An unexpectedly high number of early symptomatic UTIs was observed in the vedolizumab group, including cases caused by non‐typhoidal Salmonella species, whereas no UTIs were detected in the small comparator cohorts. Given the limited sample size and observational design, reliable estimation of relative risk was not possible.

**Conclusions:**

In this small, single‐center cohort, an unusual clustering of early symptomatic UTIs was observed among patients initiating vedolizumab. These findings are observational and hypothesis‐generating, and no causal relationship can be inferred. They highlight the importance of real‐world pharmacovigilance and the need for larger, multicenter studies to clarify whether such infection patterns reflect contextual clustering or reproducible safety signals influenced by regional epidemiological factors.

## 1 Introduction

Vedolizumab, a monoclonal antibody targeting the α4β7 integrin, is a cornerstone of treatment for moderate‐to‐severe ulcerative colitis (UC). Its mechanism—blocking lymphocyte trafficking to the gut mucosa—underpins its excellent safety profile, characterized by low rates of systemic opportunistic infection compared to anti‐TNF agents in clinical trials [1, 2]. However, real‐world evidence (RWE) is crucial for fully characterizing drug safety profiles [3]. Recent RWE studies have noted an increased incidence of non‐opportunistic infections, including UTIs, with vedolizumab use [4, 5]. Furthermore, the “gut‐urinary axis” describes a bidirectional relationship where gut microbiota and gut‐mediated immunity can influence UTI susceptibility [6, 7]. We report a striking cluster of UTIs, including infections with classic enteric pathogens, in an Iranian cohort initiating vedolizumab. This case series aims to characterize this association and propose a novel hypothesis that gut‐selective immunotherapy may, in specific regional contexts, be linked to an increased incidence of extra‐intestinal infections.

## 2 Methods

### 2.1 Study Design and Case Presentation

This was a single‐center, observational case series conducted at a tertiary referral center in Shiraz, Iran, from January 2023 to June 2024. The study cohort comprised 15 consecutive adult patients (≥ 18 years) from outpatient clinics, without selection bias beyond inclusion criteria, with moderate‐to‐severe UC (Mayo Score ≥ 6) who initiated vedolizumab therapy. Exclusion criteria included history of recurrent UTIs (≥ 2 in the past year), diabetes mellitus, urinary catheters, or known urological abnormalities. A comparator group included 30 consecutive patients initiating either infliximab (*n* = 15) or tofacitinib (*n* = 15) during the same period, with identical exclusions. All patients were prospectively followed for 12 weeks for the specific outcome of UTI. Treatment and Monitoring Patients received standard vedolizumab induction (300 mg IV at weeks 0, 2, and 6). Clinical disease activity (Mayo Score) and C‐reactive protein (CRP) were assessed at baseline and week 24 for efficacy evaluation, while the safety endpoint (UTI) was focused on the induction phase (weeks 0–12), during which infections clustered. Urine cultures were obtained from patients presenting with new symptoms suggestive of UTI (e.g., dysuria, urgency, fever); no routine urinalysis or screening cultures were performed, potentially introducing detection bias favoring symptomatic cases. A culture‐proven UTI was strictly defined as > 10^5^ colony‐forming units (CFU)/mL of a single uropathogen. No incidental asymptomatic bacteriuria was identified during the study.

### 2.2 Statistical Analysis

Descriptive statistics are presented as mean ± standard deviation or proportions. Comparative analysis of baseline characteristics was performed using ANOVA for continuous variables and chi‐square or Fisher's exact test for categorical variables, as appropriate. A two‐tailed *p* value < 0.05 was considered statistically significant. As shown in Table 1, baseline demographic and disease‐related characteristics did not differ significantly among the vedolizumab, infliximab, and tofacitinib cohorts (Table 1).

**TABLE 1:** Baseline demographic and clinical characteristics of ulcerative colitis patients treated with vedolizumab, infliximab, or tofacitinib.

Characteristic	Vedolizumab (*n* = 15)	Infliximab (*n* = 15)	Tofacitinib (*n* = 15)	*p*
Age, years (mean ± SD)	38.4 ± 12.3	40.1 ± 10.8	36.9 ± 11.5	0.75
Female sex, *n* (%)	7 (47)	6 (40)	8 (53)	0.82
Disease duration, years (mean ± SD)	5.7 ± 3.2	6.2 ± 2.9	5.1 ± 3.5	0.65
Baseline Mayo Score (mean ± SD)	8.2 ± 1.1	8.5 ± 1.3	7.9 ± 1.4	0.42
Concomitant immunomodulator^a^, *n* (%)	4 (27)	5 (33)	3 (20)	0.72
Concomitant corticosteroids, *n* (%)	6 (40)	7 (47)	5 (33)	0.81

*Note:* This table summarizes baseline demographic variables, disease characteristics, and concomitant therapies at treatment initiation. No statistically significant differences were observed among groups, indicating overall comparability of cohorts at baseline.

^a^ Immunomodulators: Azathioprine or 6‐mercaptopurine.

All patients completed the 12‐week safety follow‐up for UTI assessment. No patients had traditional UTI risk factors such as diabetes, catheters, or anatomical abnormalities. Clinical Efficacy and UTI Incidence Vedolizumab therapy was highly effective, inducing significant reductions in mean Mayo Score (from 8.2 ± 1.1 to 3.5 ± 1.2; *p* < 0.001). All infections resolved without recurrence at 6 months.

The clustering of early UTIs, including uncommon pathogens, was temporally confined to the induction phase (Table 2), supporting a potential context‐specific safety signal rather than late cumulative immunosuppression.

**TABLE 2:** Characteristics of UTI cases in the vedolizumab cohort.

Patient ID	UTI onset (week)	Pathogen	Symptoms	Treatment	Outcome
1	6	*E. coli*	Dysuria, fever, cloudy urine	Azithromycin	Resolved
2	7	*E. coli*	Dysuria, cloudy urine	Azithromycin	Resolved
3	5	Salmonella spp. (non‐typhoidal)	Dysuria, fever, cloudy urine	Azithromycin	Resolved
4	8	Salmonella spp. (non‐typhoidal)	Dysuria, cloudy urine	Azithromycin	Resolved
5	7	*E. coli*	Dysuria, fever	Azithromycin	Resolved
6	6	*E. coli*	Dysuria, cloudy urine	Azithromycin	Resolved

*Note:* No patients had traditional risk factors (e.g., diabetes, catheters). Fever present in 3/6 (50%); cloudy urine in 4/6 (67%).

Notably, two cases of non‐typhoidal Salmonella UTIs were identified; detailed case characteristics are provided in Table 2.

## 3 Discussion

This comparative case series describes an unexpected clustering of early‐onset symptomatic UTIs among patients treated with vedolizumab in a single‐center Iranian cohort. Rather than implying a causal relationship, these findings should be interpreted as observational and hypothesis‐generating.

The observed incidence of UTIs in the vedolizumab group was numerically higher than rates reported in large randomized trials such as the GEMINI studies and exceeded that observed in the small contemporaneous comparator cohorts. However, given the limited sample size, short follow‐up duration, and the absence of events in the comparator groups, reliable estimation of relative risk is not possible, and random variation or under‐detection in control groups remains a plausible explanation.

The most notable observation was the occurrence of non‐typhoidal Salmonella UTIs, an uncommon presentation even among immunocompromised individuals. Non‐typhoidal Salmonella urinary tract infections are rare and have been reported primarily as isolated case reports, even in immunocompetent adults [8]. While this finding raises the possibility of altered host–pathogen interactions, no direct mechanistic conclusions can be drawn. One speculative explanation is that modulation of gut‐specific immune trafficking by vedolizumab could influence mucosal host–microbe dynamics in a subset of patients. At present, this hypothesis lacks direct experimental or clinical validation and should be viewed solely as a framework for future investigation rather than evidence of systemic immunosuppression.

The absence of UTIs in the infliximab and tofacitinib cohorts should not be interpreted as evidence of lower true infection risk. These groups were underpowered to detect infrequent adverse events, and infections were identified only when clinically symptomatic. Consequently, the apparent contrast between treatment groups may reflect detection bias, chance, or unmeasured contextual factors rather than true biological differences.

Contextual regional factors may also have contributed to the observed clustering. Local antimicrobial resistance patterns, including high rates of fluoroquinolone‐resistant *Escherichia coli* , may increase the likelihood that infections become symptomatic and culture‐confirmed. These observations align with previous local reports documenting a high prevalence of multidrug‐resistant Escherichia coli among pathogens in central Iran [9], and recent evidence suggesting that environmental and dietary factors in the region may influence host susceptibility and microbiome composition [10], reinforcing the hypothesis that the regional context may have contributed to the observed clustering of UTIs [11]. Other potential contributors, such as environmental exposures or differences in microbiome composition, were not directly assessed in this study and therefore remain speculative.

Overall, these findings emphasize the importance of cautious interpretation of real‐world safety signals, particularly when derived from small, single‐center cohorts. They highlight the need for larger, multicenter studies with standardized infection surveillance to better characterize extra‐intestinal infection patterns associated with gut‐selective therapies.

## 4 Limitations

Our study has several key limitations. Its observational design and small sample size cannot establish causation, and the association may be influenced by unmeasured confounders not captured in our baseline comparison, such as subtle differences in disease behavior, prior antibiotic exposure, hygiene practices, or environmental exposures. Although baselines were comparable, unmeasured factors like prior antibiotic exposure or hygiene practices could confound results; propensity score matching in future cohorts could address this. With *n* = 15, the study was underpowered for subgroup analyses (e.g., by pathogen). The lack of blood cultures for febrile patients means we cannot definitively confirm or rule out bacteremia as the source of the Salmonella UTIs. While we provide local antibiogram context, we did not perform molecular characterization of the infecting strains (e.g., for virulence factors or resistance genes), which would offer deeper mechanistic insight.

## 5 Conclusion

This case series highlights an unusual clustering of early symptomatic UTIs, including rare non‐typhoidal Salmonella infections, among patients initiating vedolizumab in a specific regional context. No causal relationship can be inferred from these observations. Nevertheless, the findings underscore the value of post‐marketing surveillance and geographically diverse real‐world studies in identifying unexpected safety patterns. Further prospective investigations incorporating microbiological and epidemiological analyses are warranted to clarify whether such observations represent contextual clustering or reproducible safety signals.

## Funding

The author has nothing to report.

## Ethics Statement

The author has nothing to report.

## Consent

Written informed consent was obtained from all patients for publication of their anonymized clinical details.

## Conflicts of Interest

The author declares no conflicts of interest.

## Data Availability

The data that support the findings of this study are available from the corresponding author upon reasonable request.
